# Splenic peliosis – a potentially fatal condition which can mimick malignancy

**DOI:** 10.1186/1477-7800-4-27

**Published:** 2007-12-08

**Authors:** Gurpreet Singh-Ranger, Noleen Rajarajan, Syed Aftab, David Stoker

**Affiliations:** 1 Department of General Surgery, North Middlesex University Hospital, Sterling Way, Edmonton, London, UK

## Abstract

Isolated splenic peliosis is an extremely rare occurrence, and this disease often manifests itself with spontaneous haemoperitoneum.

We report a case where an otherwise healthy patient was found to have splenomegaly on clinical examination. On computerised tomography, a diagnosis of splenic malignancy was made, and the patient underwent a splenectomy. Histological examination gave the diagnosis of splenic peliosis, which had not been considered prior to the operation. In retrospect, splenectomy was the most prudent course of action, as the risk of spontaneous haemorrhage and fatality was eliminated. This case emphasises the need to retain an index of suspicion for this condition, even in otherwise healthy patients, and is a reminder of the usefulness of total splenectomy in the current era of minimally invasive diagnostic techniques.

## Background

Peliosis is a rare condition of unknown aetiology, characterized by the presence of multiple blood-filled cysts within the parenchyma of solid organs [1]. It tends most commonly to affect the liver, and isolated peliosis of single organs, for example the spleen, is extremely rare. Splenic peliosis has been found to be associated with chronic debilitating conditions, for example, malignancy, and with ingestion of certain medications, such as anabolic steroids. It has been reported in the literature as a consequence of its complications, namely, spontaneous rupture leading to haemoperitoneum [1].

We describe a case of asymptomatic idiopathic splenic peliosis, in which a patient presented with an incidental splenic mass on computerised tomography. This lesion had radiological features indistinguishable from a primary splenic malignancy, and the patient subsequently underwent a splenectomy. Only on histology was the lesion then confirmed to be peliosis.

## Case presentation

**Figure 1:**
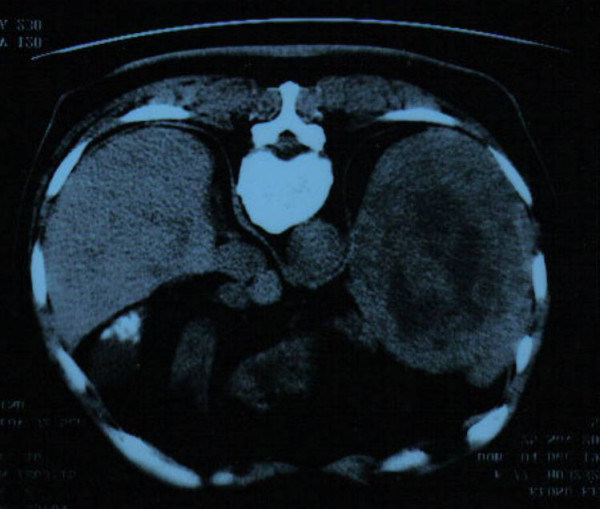
A computerised tomograph of the chest and abdomen revealing a large 13 cm × 10 cm × 13 cm hypodense, inhomogenous, non-enhancing splenic lesion.

A 78 year old Chinese gentleman presented to respiratory clinic with a 1 month history of haemoptysis. he had undergone a pneumonectomy fifty years previously in China for pulmonary tuberculosis, and was otherwise fit and well. The only abnormal finding on physical examination was splenomegaly. Bronchoscopy and cultures for mycobacteria were unremarkable. Subsequent computerised tomography of the chest and abdomen revealed a large 13 cm × 10 cm × 13 cm hypodense, inhomogenous, non-enhancing splenic lesion, with no further abnormality seen in the abdomen or chest, apart from signs consistent with previous pulmonary tuberculosis and pneumonectomy (Figure 1).

After a multidisciplinary team discussion, a diagnosis of primary splenic tumor was made, and further management in the form of a splenectomy was advised.

At operation, the spleen was found to be almost entirely replaced with a large multinodular, haemorrhagic mass. There were no other abnormal findings. A standard splenectomy was performed, and the patient was discharged uneventfully with appropriate prophylaxis.

Histopathological assessment revealed the removed spleen comprised a parafollicular zone replaced with blood-filled cystic spaces, and a central fibrinoid encapsulated area, with extensive dystrophic calcification consistent with secondary thrombosis. No amyloid, TB or fungi were found within the sample. A diagnosis of splenic peliosis was made.

On one year follow-up, the patient was healthy and well. The cause of his original haemoptysis, which ceased prior to the surgery, was never elucidated.

## Discussion

The term peliosis arises from a Greek term meaning "blackish-bluish with sugillation", and was first used by Wagner in 1861 to describe the gross appearance of lesions found on the cut surface of a liver affected by the disease [2]. Peliosis primarily affects the liver, but can also affect the spleen, lymph nodes, bone marrow, stomach, ileum and lungs [1,3]. This condition is characterised by the presence of blood-filled cysts within the affected organ which can vary in size from 1 mm to several centimeters. The pathogenesis of peliosis is not fully understood, although associations have been described with various debilitating chronic diseases, including malignancy and tuberculosis, and use of medications such as anabolic steroids and oral contraceptives [4-6]. In some cases, such as ours, no predisposing factors can be found [7,8]. It is thought that peliosis develops primarily within organs belonging to the mononuclear phagocyte system, and that these lesions represent venous malformations which manifest under locally altered intravascular pressure conditions, which may be congenital or acquired in origin [3]. Some reports have described the presence of immune-complex deposits, and abundant reticulendothelial cells in the vicinity of peliotic lesions [9,10]. Etzion et al reported a case of atraumatic rupture of the spleen associated with both peliosis and haemophagocytic syndrome, a clinical syndrome resulting from enhanced macrophage activity [11]. Microscopic analysis of the spleen revealed generalised splenic peliosis, in association with excessive macrophages around the blood cavities and red pulp. These findings have led to speculation of an immune-complex mediated basis for the condition. Gugger et al using immunochemical staining, detected local deposits of immunoglobulin G and activated complement C3 in the vicinity of peliotic lesions [10], which supports this hypothesis.

Isolated splenic peliosis is extremely rare [3], occurring in less than 1% of cases at autopsy [12]. Complications arise when peliotic lesions rupture. This can occur spontaneously, most likely due to intrinsic pressure effects, or as a consequence of extrinsic trauma. The resulting intraperitoneal haemorrhage can be fatal [1,6].

Our case is unusual, as the patient described was otherwise fit and well, with no evidence of chronic disease. Neither was he taking any medications associated aetiologically with peliosis. Consequently, the finding of an isolated splenic mass posed a diagnostic and therapeutic challenge. Incidental splenic masses are sometimes encountered in patients undergoing imaging for other diseases [13], and primary vascular tumours and lymphoma are amongst the commonest differential diagnoses. Fine needle aspiration of the spleen [sFNA] has been reported to be useful in establishing a diagnosis in cases of idiopathic splenomegaly, with or without radiological guidance [13]. This technique can however, lead to bleeding in 1 – 10% of cases, and also carries a concomitant risk of splenectomy [14-16]. Recently, laparoscopic splenic biopsy has attracted interest in elucidating the cause of splenomegaly of unknown origin, but the safety and effectiveness of this technique is yet to be clarified [17]. Although splenic peliosis is termed a "benign condition", serious complications such as spontaneous haemorrhage and fatality can occur. The risk of these events occurring has not been quantified, but it is clear that even small peliotic blebs can rupture and prove to be fatal [18]. Moreover, the safety of sFNA and image-guided splenic biopsy in these circumstances has not been evaluated. Some useful information is gained from reported series of invasive diagnostic splenic interventions. In one group, the majority of emergency splenectomies and bleeding episodes occurred in patients with an underlying vascular tumour of the spleen [15].

In our case, histology revealed almost complete replacement of the spleen by peliotic lesions, and we believe a policy of biopsy-guided diagnosis and conservative management would have exposed the patient to the unacceptable risk of suffering from spontaneous haemorrhage. Our patient therefore underwent a splenectomy, which we believe in retrospect, was the best course of action. Splenectomy offers the advantage of a definitive histological diagnosis and the complete elimination of risk of spontaneous haemorrhage. It does however, result in the lifelong requirement of antibiotic prophylaxis.

There are no published guidelines on managing patients with suspected splenic peliosis, and as this condition is so rare, it is also frequently overlooked as a potential differential diagnosis in cases of idiopathic splenomegaly. We feel clinicians should retain a high index of suspicion for the presence of this disease, and be aware of the potentially fatal consequences of missed diagnosis. In cases where peliosis enters the differential diagnosis of a splenic mass, total splenectomy should be considered as the most prudent course of action.
